# The Relationship between Problematic Facebook Use and Early Maladaptive Schemas

**DOI:** 10.3390/jcm9123921

**Published:** 2020-12-03

**Authors:** Andrzej Cudo, Dorota Mącik, Mark D. Griffiths, Daria J. Kuss

**Affiliations:** 1 Institute of Psychology, The John Paul II Catholic University of Lublin, al. Racławickie 14, 20-950 Lublin, Poland; dmacik@kul.pl; 2 International Gaming Research Unit, Nottingham Trent University, 50 Shakespeare St, Nottingham NG1 4FQ, UK; mark.griffiths@ntu.ac.uk (M.D.G.); daria.kuss@ntu.ac.uk (D.J.K.)

**Keywords:** Facebook use, problematic Facebook use, problematic social media use, problematic social networking use, early maladaptive schemas

## Abstract

Facebook is an increasingly popular online social media platform for communication, entertainment, and information exchange. Previous studies have shown the relationship between problematic Facebook use (PFU) and mental health problems. Additionally, previous studies have reported associations between maladaptive cognitive schemas and mental health problems. However, little is known about their impact on problematic behavior associated with Facebook use. Consequently, the present study investigated the relationship between PFU and early maladaptive schemas (EMSs) among Facebook users. The study comprised 619 Facebook users (568 females; age range from 18 to 30 years; *M* = 21.34, *SD* = 2.41 years). The severity of PFU was assessed using the Facebook Intrusion Scale, and EMS dimensions were assessed using the 90-item Young Schema Questionnaire (YSQ–S3). The findings showed a positive relationship between PFU and EMSs, such as insufficient self-control/self-discipline and approval seeking. PFU was negatively associated with EMSs, such as social isolation/alienation and self-sacrifice schemas. Additionally, the findings showed that EMSs, as well as Facebook use characteristics, such as the time spent using Facebook per week, using Facebook apps, and number of friends on Facebook contributed to explaining the variance in PFU scores. These findings may contribute to a better understanding of mechanisms related to the development of PFU, which are associated with cognitive schemas. Additionally, the results may be useful in developing more effective methods of prevention and treatment of this problematic behavior.

## 1. Introduction

Problematic Facebook use (PFU) has been defined as excessive engagement in Facebook activities causing problems in everyday social functioning [1]. Andreassen and Pallesen [2] described such behavior as “being overly concerned about SNSs (Social Networking Sites), to be driven by a strong motivation to log on to or use SNSs, and devote so much time and effort to SNSs that it impairs other social activities, studies/job, interpersonal relationships, and/or psychological health and well-being” (p. 4054). Additionally, Griffiths [3,4,5] has asserted that all behavioral addictions (including addictions to social media) comprise six components: salience, mood modification, tolerance, withdrawal, conflict and relapse. Griffiths [5] asserts that, in order to recognize such behavior as addiction, all six listed components must be present. However, problematic behavior may still be present even when some of these consequences are absent. Additionally, it should be noted that there is no official diagnosis of PFU in any diagnostic manuals.

The prevalence rates of PFU have been heterogeneous and different studies report various rates. However, some research has reported that the prevalence rate of PFU does not exceed 10% of Facebook users (e.g., polythetic scoring [6]: 4.5%–8.4%; monothetic scoring: 0.6%–1.7% [6]; polythetic scoring: 6.2% [7]; monothetic scoring: 2.5% [7]; 4.88% [8]). Additionally, female users appear to be more vulnerable to PFU than male users [7,8,9,10,11]. For example, Cudo et al. [8] reported that females had much higher levels of PFU (6.4%) compared to males (3.1%). Additionally, other studies [7,10] have reported a positive relationship between PFU and female gender.

Previous research [7,12,13,14] has shown that PFU is associated with negative mental health consequences (e.g., depression, anxiety, insomnia, and stress), and difficulties in interpersonal relationships. Additionally, previous findings have indicated that negative parent and peer attachment were associated with PFU [15,16,17]. Negative attachment has been associated with poor mental health and interpersonal relationship problems, difficulties in self-representations, and maladaptive emotion regulation [18,19]. Additionally, previous research [20,21,22] has shown that early maladaptive schemas (EMSs) are mediators between negative attachment and mental health problems. Taking into account that EMSs are “extremely stable and enduring themes that develop during childhood and are elaborated upon throughout an individual’s lifetime” ([23], p. 9) and EMSs are a cognitive–emotional representation of the knowledge individuals have about themselves and the world they live in [23], they can be essential in understanding the development of problematic behaviors, such as PFU. However, there are very few studies examining the relationship between problematic behaviors associated with internet use and such schemas [24,25]. Additionally, they only focus on general problematic internet use and avoid other problematic behaviors related to internet use, such as PFU. Consequently, the aim of the present study was to investigate EMSs related to PFU and to identify which individual beliefs are associated with this problematic behavior. Understanding these relationships will aid in the prevention and treatment of PFU, especially in the context of behavior modification in cognitive behavioral therapy.

According to Young, Klosko and Weishaar [26], EMSs develop on the basis of unmet needs and traumatic experiences during childhood. Additionally, these needs and experiences with significant figures (e.g., parents, siblings, peers) are combined with the child’s emotional temperament. For example, children, whose parents neglect them and their basic psychological needs (e.g., attention, acceptance, and unconditional love) are unmet, may develop the strong and stable belief “I am unimportant”. Consequently, these kinds of negative beliefs dominate thoughts and feelings, and may lead to negative emotions and dysfunctional thoughts. EMSs also regulate how individuals view themselves as well as their interpersonal relationships with others [23]. Young [27] described 18 early maladaptive schemas, which can be categorized within five domains. However, Bach, Lockwood and Young [28] modified this classification and postulated only four domains (see Table 1). Additionally, previous research has shown that EMSs are related to psychological problems, including depression [29,30,31,32], anxiety [31,32], social phobia [33], and obsessive–compulsive disorder [34,35]. EMSs are also associated with personality disorders, including dependent personality disorder, borderline personality disorder, and antisocial personality disorder [36,37,38,39]. Previous research has also shown that EMSs are related to substance use [40,41], and behavioral addictions, such as problematic gambling [24,42], food addiction [24,43], compulsive sexual behavior [44], and problematic smartphone use [45]. 

Additionally, Aloi et al. [24], and Shajari, Sohrabi, and Jomehri [25] demonstrated there is a relationship between problematic internet use and EMSs. More specifically, Aloi et al. [24] conducted a study comprising 1075 university students to determine the difference between low and high problematic internet use groups across the EMSs dimensions. Based on logistic regression, they reported that younger age and higher scores on the EMSs dimensions, such as that of disconnection and rejection, responsibility accountability and standards, and impaired limits were associated with high problematic internet use. Shajari, Sohrabi and Jomehri [25] conducted a study comprising 195 university students and reported a positive relationship between problematic internet use and all EMSs. However, they only calculated correlation coefficients between EMSs and problematic internet use separately for each EMS. Consequently, they ignored the interrelationships between EMSs. Taken together, these studies [24,25] appear to indicate that EMSs can be important contributors to problematic behaviors associated with general internet use. However, taking into account the differences between subtypes of these problematic online behaviors, such as problematic general internet use, problematic videogame playing, and problematic Facebook use [8,46,47,48], it is essential to verify EMSs that may be related to the development of these problematic online behavior subtypes.

Consequently, the present study investigated the relationship between PFU and EMSs. Based on previous research concerning the relationship between EMSs and problematic internet use [24,25] and the positive relationship identified between the time spent using Facebook per week and PFU [8], it was hypothesized that PFU would be positively associated with EMSs. Previous research [6,7,49,50] has reported a positive relationship between narcissism and PFU. Additionally, Carr and Francis [38] reported that the narcissistic personality trait is positively associated with the entitlement/grandiosity schema and negatively associated with self-sacrifice and defectiveness/shame schemas. Bach et al. [36] reported that narcissism was positively related to the entitlement/grandiosity schema and negatively related to failure to the achieve schema. Nordahl, Holthe and Haugum [51] reported a positive relationship between narcissistic personality traits and EMSs such as vulnerability to harm or illness, emotional inhibition and insufficient self-control/self-discipline. Consequently, taking into account the relationship between narcissism and PFU [6,7,49,50], and the relationship between narcissistic personality traits and EMSs [36,38,51], it was assumed that PFU would be positively associated with entitlement/grandiosity, vulnerability to harm or illness, emotional inhibition, and insufficient self-control/self-discipline schemas. Additionally, it was assumed that PFU would be negatively associated with self-sacrifice, defectiveness/shame, and failure to achieve schemas.

Moreover, it should be noted that social media such as Facebook allows users to post, comment, and like content [52]. In this context, Reich et al. [53] reported that users who received many “likes” had a higher level of belongingness and self-esteem compared to users who did not receive any “likes”. Additionally, Scissors et al. [54] reported that low self-esteem, high self-monitoring, and a high number of friends were associated with feeling bad when Facebook users did not receive enough “likes”. Other studies [55,56] have also shown that “likes” are important for the users’ feeling of belonging and well-being. Additionally, Vaillancourt-Morel et al. [57] showed that "like"-seeking behavior was positively associated with PFU. In this context, it was assumed that Facebook users who believe that their value depends on positive social approval expressed by “likes” from other Facebook users may have a higher probability of PFU. Taking into account EMSs, it was assumed that an approval seeking schema would be positively associated with PFU.

This assumption is also supported by other studies, which indicate that attachment anxiety predisposes Facebook users to sensitivity concerning social feedback [57,58]. In this context, Hart et al. [58] postulated that attachment anxiety predisposes Facebook users to engage in attention-seeking social media behavior. Additionally, Vaillancourt-Morel et al. [57] showed "like"-seeking behavior partially mediated the relationship between attachment anxiety and PFU. Taking into account that Mącik [59] showed that anxious-ambivalent attachment was positively associated with abandonment, emotional deprivation, and approval seeking schemas, it was assumed that an approval seeking schema is probably an essential schema in understanding the mechanism of PFU development. Additionally, these studies [59] may also indicate possible links between PFU and EMS, such as abandonment and emotional deprivation.

Taken together, the results of the aforementioned previous studies suggest that PFU would be positively associated with some EMSs, such as entitlement/grandiosity, vulnerability to harm or illness, emotional inhibition, insufficient self-control/self-discipline, approval seeking, abandonment, and emotional deprivation schemas. Additionally, it was also assumed that PFU may be negatively associated with EMSs, such as self-sacrifice, defectiveness/shame, and failure to achieve schemas. Consequently, the present study aimed to verify these assumptions.

Moreover, taking into account previous research [8] showing the relationship between PFU and Facebook use characteristics, such as the time spent using Facebook per week, using Facebook apps, the number of friends on Facebook, and the number of years using Facebook were also included in the present study. In this context, the aim of the present study was to verify the extent to which Facebook use characteristics and EMSs contribute to explaining PFU.

## 2. Materials and Methods

### 2.1. Participants

The study comprised 692 individuals selected from 1365 individuals who completed an offline survey. Taking into account the differences between PFU and PVG [8,48] and to ensure group homogeneity, individuals who played videogames were excluded from the analysis. In this context, Dredge and Chen [60] found differences between gamers and non-gamers in relation to their social media use and reported that gamers (i) engaged in more socializing on social media, (ii) spent more time on social media, and (iii) had higher levels of social media addiction. The criterion for exclusion of selected participants was based on whether they had played videogames in the past year. Consequently, 673 gamers (391 female gamers) were excluded from analyses. Additionally, 73 participants were excluded due to missing data and outliers. Consequently, the final sample comprised 619 participants (568 females) aged 18–30 years (*M* = 21.34 years, *SD* = 2.41). All participants were volunteers, received no monetary reward for participation, and were Facebook users. The participants were from six voivodships (administrative regions) of Poland (Małopolskie, Mazowieckie, Kujawsko-Pomorskie, Lubelskie, Lubuskie and Wielkopolskie). The study was conducted in accordance with the Declaration of Helsinki, and was approved by the Institute of Psychology’s ethics committee (John Paul II Catholic University of Lublin). It should also be noted that the present study is a part of a larger research project on psychological aspects of problematic Facebook use and problematic video gaming, such as the early maladaptive schemas and multidimensional aspects of impulsivity among young Polish adults. Taking into account the breadth and consistency of the issues, only the variables concerning the relationship between the early maladaptive schemas and PFU among Polish Facebook users are examined in the present study. Additionally, the relationship between the multidimensional aspects of impulsivity and problematic behaviors was reported in another paper [61]. The dataset from the present study is available from the John Paul II Catholic University of Lublin repository database (accession link: http://hdl.handle.net/20.500.12153/1380).

### 2.2. Materials

Problematic Facebook Use: PFU was assessed using the eight-item Facebook Intrusion Scale [8]. Each item (e.g., “The thought of not being able to access Facebook makes me feel distressed”) is rated on a Likert scale ranging from 1 (strongly disagree) to 7 (strongly agree). The scores range from 8 to 56 points, and higher scores reflect a greater intensity of PFU. In the present study, the Cronbach’s alpha was very good (0.85).

Early Maladaptive Schemas: Early maladaptive schemas were assessed using the 90-item Young Schema Questionnaire (YSQ–S3) [62,63]. Each item is rated on a Likert scale ranging from 1 (completely untrue of me) to 6 (describes me perfectly). For each schema (see Table 1), the minimum score is 5 and the maximum score is 30. Higher scores reflect a greater intensity of the schema. The schema subscales have good psychometric properties, with Cronbach’s alphas ranging from 0.61 to 0.87 in the present study (see Table 1).

Demographic variables and frequency of use: Participants also provided information concerning socio-demographic factors (i.e., age and gender) and the time spent using Facebook per week. Additionally, the participants were asked whether they used Facebook apps on their smartphones. The participants were also asked to indicate their number of friends on Facebook (using a nine-point scale, from 1 [0–100 friends] to 9 [over 800 friends]) and the number of years they had used Facebook.

### 2.3. Statistical Analysis

The descriptive statistics, such as means and standard deviations for all the variables, as well as Spearman correlation coefficients between the variables, were calculated. In order to verify the relationship between PFU, Facebook use characteristics, and early maladaptive schemas, hierarchical regression was used. In the first step, age and gender were entered into the regression equation, and in the second step, the characteristics of Facebook use, such as the time spent using Facebook per week, using Facebook apps, the number of friends on Facebook and the number of years using Facebook, were included into the regression model. In the third step, the early maladaptive schemas were included. Taking into account the violation of assumption of homoscedasticity (Breusch-Pagan/Cook-Weisberg test for heteroscedasticity; χ^2^_(df = 1)_ = 32.43; *p* < 0.001) [64,65], the regression equation with robust standard errors was used. Based on Ramsey’s [66] regression specification-error test (RESET; *F*_(3, 591)_ = 0.74; *p* = 0.526), analysis showed that there were no omitted variables. The variable inflation factor (VIF) was used to assess multicollinearity. Results indicated that all values for the independent variables were below the multicollinearity threshold of 10. Consequently, there was no violation in the assumption of multicollinearity [67]. The descriptive statistics and regression analyses were conducted using Stata 14.

## 3. Results

The descriptive statistics, such as mean and standard deviation and correlation coefficients are presented in Table 1. The results showed that PFU was positively associated with the number of friends on Facebook, the number of Facebook hours per week, the number of years using Facebook, and using Facebook apps on the smartphone. Additionally, there was a positive relationship between PFU and almost all early maladaptive schemas, except emotional deprivation, social isolation/alienation, self-sacrifice, emotional inhibition, and unrelenting standards. Detailed findings are presented in Table 2.

According to a hierarchical regression analysis, in the first step (*R*^2^ = 0.01; *F*_(2, 616)_ = 2.79; *p* = 0.062), gender was a significant predictor of PFU. Entering Facebook use characteristic variables in the second step resulted in a statistically significant increase in the explained variance (*R*^2^ = 0.19; *F*_(6, 612)_ = 21.26; *p* < 0.001). Examination of the beta weights indicated that the time spent using Facebook per week, using Facebook apps, and the number of friends on Facebook had significant positive beta weights, whereas gender became non-significant. The third step, including early maladaptive schemas, led to increasing the explained variance (*R*^2^ = 0.30; *F*_(24, 594)_ = 11.83; *p* < 0.001). Additionally, insufficient self-control/self-discipline and approval-seeking had significant positive beta weights, whereas social isolation/alienation and self-sacrifice had significant negative beta weights. Moreover, Facebook use characteristics were still statistically significant. Detailed findings are presented in Table 3.

## 4. Discussion

The present study’s main aim was to investigate the relationship between PFU and EMSs. Additionally, the present study investigated how much EMSs contribute to explaining the variance in PFU scores in addition to the Facebook use characteristics. The findings showed that PFU was positively related to insufficient self-control/self-discipline and approval seeking schemas. There was a negative relationship between PFU and EMSs, such as with social isolation/alienation and self-sacrifice schemas. Additionally, the findings showed that EMSs, in addition to Facebook use characteristics, contributed to explaining the variance in PFU scores. In the context of Facebook use characteristics, there was a positive relationship between time spent using Facebook per week, using Facebook apps, the number of friends on Facebook, and PFU. However, the number of years using Facebook was not related to this PFU.

As hypothesized, there were positive and negative relationships between specific EMSs and PFU. More specifically, there was a positive relationship between the insufficient self-control/self-discipline schema and PFU. This result may indicate that pervasive difficulty in maintaining an appropriate level of self-control and frustration tolerance to achieve an individual’s goals [23] may contribute to increased PFU. In this context, PFU may also be associated with difficulty in self-control to restrain the excessive expression of an individual’s emotions and impulses. It should be noted that this schema occurs when parents do not maintain rules and discipline in raising their children (see [23]). These findings are in line with previous research [68,69], indicating the negative relationship between self-control traits and PFU. Consequently, it can be assumed that this EMS formed in childhood may lead to difficulties with perseverance, and that difficulties with delayed gratification contribute to the development of PFU. However, further research is needed to answer the question of whether this schema is also associated with other subtypes of problematic internet use.

Additionally, the results of the present study showed a weak correlation between PFU and entitlement/grandiosity. However, there was no relationship between the entitlement/grandiosity schema and PFU in the regression model. Additionally, the approval seeking schema was related to PFU. It should be noted that the approval seeking schema is part of the same EMS dimension as the entitlement/grandiosity schema (see Table 1). More specifically, the entitlement/grandiosity schema is associated with the belief that the individual is someone special, privileged, better, and therefore disregards the needs or opinions of others. In contrast, the approval seeking schema is associated with a strong need for social approval, often at the expense of an individual’s own needs [23]. In this context, Bach, Lockwood and Young [28] pointed out that there are two routes to developing narcissism. The first route is associated with the family system in which children’s basic emotional needs are ignored, and children are used to satisfy their parents’ egoistic needs [70,71]. Consequently, children overcompensate for basic feelings of inferiority, loneliness, and lack of gratification through a grandiose self-image [70,71].

The second route is associated with the family system in which children get everything they want and when they want it, and parents spoil them excessively [70,71]. Consequently, children may believe that they are entitled to special rights, and other individuals have to accept it. Taking into account the first type of narcissism development, children with unmet basic emotional needs may develop a strong need for social approval to meet their emotional needs. In this context, it can be assumed that PFU may be associated with the first type of family system. This assumption is supported by Casale and Fioravanti’s [72] research, which indicated that the relationship between grandiose narcissism and PFU was fully mediated by the need to belong and the need for admiration. However, further research is needed to verify this assumption.

Another explanation for these relationships may be related to the search for social approval by Facebook users. More specifically, Facebook users with an approval-seeking schema may consider Facebook as a place where they can receive approval from other users by “likes” or comments [57,58]. Such users may also become more involved in Facebook because “likes” and comments may satisfy their emotional needs. Consequently, they may be more vulnerable to PFU. However, this assumption should be verified in future studies.

There was a negative relationship between the self-sacrifice schema and PFU. More specifically, the lack of a tendency to take care of others instead of themselves [23] may contribute to increased problems with Facebook use. This finding is complemented by results concerning the relationship between the approval seeking schema and PFU in the present study. Additionally, PFU was negatively associated with the social isolation/alienation schema. This result may indicate that a weak belief that the individual is completely different from other people and not fit for society [23] may be associated with problems concerning Facebook use. One possible explanation for this finding may be related to narcissism. In this context, Zeigler-Hill et al. [73] reported a negative relationship between social isolation/alienation and narcissism dimensions, such as leadership/authority and superiority/arrogance. Another possible explanation may be related to the online relationship via Facebook. In this context, Tang et al. [74] reported that PFU was positively associated with both online relationships (intimacy and information disclosure) and social support (information, affectionate and companionship support). Taking into account the results of Tang et al.’s [74] study, it can be assumed that individuals with a large number of online relationships and strong social support associated with these relationships may believe that they are part of a social group. Consequently, individuals with PFU who spend a lot of time on Facebook and whose social activities are on Facebook may feel that they are part of a social group. Another possible explanation for the negative relationship between the self-sacrifice schema, social isolation/alienation schema, and PFU may be related to the suppression effect. More specifically, these relationships were only observed when Facebook use characteristics were controlled for. Consequently, controlling characteristics of Facebook use, such as number of friends on Facebook, number of hours using Facebook per week, number of years using Facebook, and Facebook smartphone app use allowed for these relationships to be observed. However, further research is needed to verify the aforementioned assumptions.

Additionally, there was no relationship between PFU and the defectiveness/shame and failure to achieve schemas in the regression model. Consequently, it can be assumed that these schemas are not related to PFU. More specifically, the belief that the individual is defective and unlovable at some fundamental level (defectiveness/shame schema; [23]) was not related to PFU. Similarly, the belief that it is not possible to achieve as much as others due to poorer competence/ability (failure to achieve schema; [23]) was not related to this type of problematic behavior. In this context, it should be noted that Facebook users have control over self-presentation on this social networking site [75,76]. Consequently, Facebook users can choose which self-information they want to publish on their Facebook profile and which information they want to skip. Additionally, they may also create a false self-presentation on Facebook [77]. In this context, taking into account the relationship between PFU and the social approval schema found in the present study, it might be that Facebook users with PFU are more likely to create a virtual representation of themselves on Facebook in order to seek social approval than to hide their failures or defects. It should be noted that the social approval schema is more than just taking into account social approval in behavior. An individual’s feelings and thoughts about themselves are primarily dependent upon the reactions of others rather than on their natural inclinations [23]. Moreover, this schema can lead to many negative consequences in an individual’s life. Further research is needed to verify the assumption concerning creating a virtual representation of themselves on Facebook in order to seek social approval rather than to hide their perceived failures or defects.

There was no relationship between emotional inhibition, vulnerability to harm or illness schemas, emotional deprivation, abandonment, and PFU. More specifically, the belief that it is necessary for an individual to suppress their own spontaneous emotions and impulses is bad (emotional inhibition schema; [23]) was not related to PFU. Similarly, the belief that something terrible could happen at any time (vulnerability to harm or illness schema; [23]) was not associated with this problematic behavior in the regression model. The belief that emotional needs are not important and will not be met by others (emotional deprivation schema; [23]) and the belief that other individuals will be unable to provide emotional support because they will leave sooner or later (abandonment schema; [23]) were not associated with PFU. These results may indicate that these schemas are not related to PFU. Additionally, the results showed a positive association between PFU and Facebook use characteristics, such as the time spent using Facebook per week, using Facebook apps, and number of friends on Facebook. However, PFU was not related to the number of years using Facebook. These findings are similar to previous research [8], indicating similar relationships between the variables. In this context, Cudo et al. [8] noted that individuals with PFU may transfer their social relationship from offline to the online social networking platform as they increase their time spent on Facebook and use Facebook not only on a computer but also on a smartphone.

Regarding the present findings, it is necessary to take into account some limitations. Firstly, the study group was selected from the Polish population. Consequently, caution should be exercised when generalizing the results to other countries and cultural contexts. Secondly, the study utilized self-report methods and was cross-sectional. Consequently, causal relationships cannot be determined. Accordingly, stronger conclusions cannot be provided given the cross-sectional and self-report nature of this study. Further longitudinal research is needed to confirm the results more robustly. Thirdly, taking into account that PFU is only one example of problematic social media use [78], the generalization of results to other social media platforms cannot necessarily be made. Fourth, most of the participants were female. Therefore, caution should be exercised in generalizing the results to males. Fifth, the present study included Facebook users who had not played videogames in the past year. Consequently, caution should be exercised when generalizing the results to Facebook users who play videogames.

The results of the present study showed an association between PFU and EMSs, including the insufficient self-control/self-discipline, approval seeking, social isolation/alienation, and self-sacrifice schemas. These findings may be important for understanding the mechanisms underlying PFU development. Consequently, they can contribute to the development of treatment and prevention of PFU. More specifically, based on the schema therapy framework [23], it can be assumed that changing the cognitive patterns connected with the aforementioned schemas may lead to the suppression of problematic behavior associated with Facebook use. However, further research is needed to fully confirm this conjecture.

## Figures and Tables

**Table 1:** Description of Early Maladaptive Schemas.

Dimension (Bach, Lockwood and Young, 2018)	Dimension Description	Schemas	Description of Schemas	Cronbach’s Alpha
Disconnection and Rejection	The schemas connected to general belief that the needs for security, stability, care and acceptance will never be met	Emotional Deprivation	The belief that emotional needs are not important and will not be met by others.	0.80
		Defectiveness/Shame	The individual’s belief that there is something wrong with them that they have some permanent defect in them.	0.87
		Mistrust/Abuse	The individual’s belief that others can hurt, abuse, cheat or humiliate them.	0.83
		Social Isolation/Alienation	The belief that the individual is completely different from other individuals and does not belong to society.	0.85
		Emotional inhibition	The belief that it is necessary to suppress spontaneous emotions and impulses.	0.79
		Pessimism/Negativism	The belief that everything will turn out badly.	0.81
Impaired autonomy and Performance	The schemas are associated with dependence on others, feeling insecure and suffer from a lack of self-determination.	Dependence/Incompetence	The individual’s belief that they need considerable help from others to manage everyday responsibilities.	0.74
		Vulnerability to harm or illness	The belief that a negative incident can happen at any time and that it will not be possible to prevent it.	0.77
		Abandonment	The belief that other individuals will be unable to provide emotional support because they will sooner or later leave.	0.82
		Enmeshment	Excessive emotional over-involvement and closeness with other individual or individuals.	0.72
		Failure to achieve	The belief that it is not possible to achieve as much as others due to poorer competence/ability.	0.86
		Subjugation	The individual’s belief that they must submit to the will of others because otherwise they will face some unpleasant consequences.	0.74
Excessive Responsibility and Standards	The schemas are associated with high, often impossible to meet expectations and a perfectionist approach to achievements.	Self-sacrifice	A desire to satisfy the others’ needs so as not to hurt them	0.75
		Unrelenting standards	The individual’s belief that they must meet unrealistic and high standards.	0.74
		Self-punitiveness	The belief that every mistake deserves severe punishment.	0.78
Impaired Limits	The schemas associated to lack of responsibility, unstable self-assessment, inability to achieve distant goals.	Entitlement/Grandiosity	The belief of being someone special who has special privileges and can do and say what they want, whether it is acceptable to others or not.	0.61
		Insufficient self-control/self-discipline	The belief that self-discipline is unimportant.	0.76
		Approval seeking	The belief that an individual’s value depends on positive social approval.	0.80

**Table 2:** Mean values, standard deviations and correlation coefficients between analysed variables (*N* = 619).

Variables		*M*	*SD*	[1]	[2]	[3]	[4]	[5]	[6]	[7]	[8]	[9]	[10]	[11]	[12]	[13]	[14]	[15]	[16]	[17]	[18]	[19]	[20]	[21]	[22]	[23]	[24]
[1] Problematic Facebook use		21.60	9.36																								
[2] Number of friends on Facebook		5.22	2.37	0.26 ***																							
[3] Number of hours using Facebook per week		22.24	26.44	0.37 ***	0.14 **																						
[4] Number of years using Facebook		7.15	2.20	0.12 **	0.22 ***	-0.05																					
[5] Facebook smartphone apps use		0.95	0.22	0.20 ***	0.14 **	0.12 **	0.03																				
**Early maladaptive schemes**	[6] Emotional Deprivation	9.42	4.80	0.06	−0.03	0.05	−0.06	0.02																			
	[7] Abandonment	14.90	6.28	0.17 ***	0.03	0.01	0.10 *	0.03	0.37 ***																		
	[8] Mistrust/Abuse	12.73	5.67	0.18 ***	0.05	0.06	0.08	0.02	0.49 ***	0.64 ***																	
	[9] Social Isolation/Alienation	12.10	5.75	−0.02	−0.18 ***	−0.08	−0.03	−0.04	0.52 ***	0.49 ***	0.59 ***																
	[10] Defectiveness/Shame	9.31	5.24	0.11 **	−0.07	0.06	−0.06	0.01	0.64 ***	0.43 ***	0.50 ***	0.60 ***															
	[11] Failure to Achieve	11.68	5.65	0.13 **	−0.05	0.01	0.01	0.01	0.46 ***	0.54 ***	0.52 ***	0.60 ***	0.60 ***														
	[12] Dependence/Incompetence	10.55	4.68	0.20 ***	0.01	0.07	0.02	−0.03	0.41 ***	0.56 ***	0.51 ***	0.52 ***	0.57 ***	0.66 ***													
	[13] Vulnerability to Harm or Illness	11.47	5.41	0.21 ***	0.04	0.06	0.08 *	−0.01	0.35 ***	0.58 ***	0.57 ***	0.49 ***	0.47 ***	0.55 ***	0.61 ***												
	[14] Enmeshment	9.98	4.50	0.19 ***	0.07	0.13 **	0.02	0.01	0.34 ***	0.47 ***	0.44 ***	0.41 ***	0.44 ***	0.47 ***	0.53 ***	0.49 ***											
	[15] Subjugation	10.63	4.73	0.24 ***	0.04	0.10 *	0.06	0.04	0.52 ***	0.58 ***	0.60 ***	0.54 ***	0.58 ***	0.65 ***	0.69 ***	0.57 ***	0.58 ***										
	[16] Self-sacrifice	15.77	5.18	0.05	0.16 ***	0.01	0.12 **	0.06	0.22 ***	0.42 ***	0.42 ***	0.24 ***	0.16 ***	0.31 ***	0.23 ***	0.29 ***	0.31 ***	0.40 ***									
	[17] Emotional Inhibition	12.03	5.47	0.05	−0.17 ***	0.01	−0.02	0.01	0.46 ***	0.43 ***	0.53 ***	0.65 ***	0.57 ***	0.53 ***	0.48 ***	0.41 ***	0.41 ***	0.54 ***	0.24 ***								
	[18] Unrelenting Standards	15.26	5.38	0.05	0.08	−0.07	0.13 **	0.02	0.26 ***	0.47 ***	0.44 ***	0.40 ***	0.25 ***	0.30 ***	0.30 ***	0.38 ***	0.33 ***	0.35 ***	0.49 ***	0.41 ***							
	[19] Entitlement/Grandiosity	14.18	4.53	0.13 **	0.15 ***	−0.04	0.12 **	0.06	0.24 ***	0.36 ***	0.45 ***	0.33 ***	0.21 ***	0.26 ***	0.24 ***	0.34 ***	0.29 ***	0.33 ***	0.38 ***	0.33 ***	0.49 ***						
	[20] Insufficient Self-Control/Self-Discipline	14.25	5.24	0.24 ***	0.09 *	0.02	0.16 ***	0.04	0.32 ***	0.48 ***	0.48 ***	0.38 ***	0.35 ***	0.53 ***	0.53 ***	0.51 ***	0.39 ***	0.54 ***	0.33 ***	0.40 ***	0.29 ***	0.49 ***					
	[21] Approval Seeking	16.25	5.72	0.28 ***	0.15 ***	0.01	0.19 ***	0.06	0.19 ***	0.56 ***	0.46 ***	0.31 ***	0.25 ***	0.37 ***	0.38 ***	0.39 ***	0.31 ***	0.43 ***	0.37 ***	0.30 ***	0.51 ***	0.52 ***	0.55 ***				
	[22] Pessimism/Negativism	14.00	5.80	0.19 ***	0.09 *	0.05	0.14 ***	0.01	0.36 ***	0.67 ***	0.67 ***	0.48 ***	0.43 ***	0.58 ***	0.60 ***	0.76 ***	0.47 ***	0.61 ***	0.42 ***	0.45 ***	0.46 ***	0.40 ***	0.56 ***	0.51 ***			
	[23] Self-Punitiveness	11.93	4.95	0.09 *	0.06	0.06	0.04	−0.04	0.32 ***	0.48 ***	0.46 ***	0.41 ***	0.41 ***	0.51 ***	0.46 ***	0.50 ***	0.41 ***	0.47 ***	0.40 ***	0.41 ***	0.56 ***	0.33 ***	0.39 ***	0.37 ***	0.57 ***		
[24] Age		21.34	2.41	0.01	−0.10 *	0.01	0.25 ***	−0.05	−0.03	−0.12 **	−0.12 **	0.01	−0.06	−0.06	−0.08	−0.02	−0.06	−0.03	−0.03	−0.02	0.05	−0.01	0.01	−0.03	−0.04	−0.03	
[25] Gender		0.08	0.28	−0.08 *	−0.11 **	−0.05	−0.13 **	−0.01	0.10 *	−0.08 *	−0.02	0.05	0.05	−0.07	0.01	−0.04	0.05	−0.03	−0.09 *	0.06	0.03	0.06	0.01	−0.01	−0.06	0.01	0.07

*** *p* < 0.001, ** *p* < 0.01, * *p* < 0.05. Note: gender: 0 = female, 1 = male; Facebook smartphone apps use: 0 = No, 1 = Yes; means of Facebook smartphone apps use and gender described the percentage of users of the Facebook apps and the percentage of male, respectively.

**Table 3:** Results of hierarchical regression.

Variables		Step 1			Step 2			Step 3		
		B	SE	β	B	SE	β	B	SE	β
Age		−0.03	0.14	0.01	0.11	0.14	0.03	0.15	0.14	0.04
Gender		−2.01	1.21	−0.08 *	−1.40	1.06	−0.04	−1.68	1.03	−0.05
Number of Facebook friends					0.77	0.16	0.20 ***	0.60	0.16	0.15 ***
Number of hours per week on Facebook					0.11	0.01	0.30 ***	0.10	0.01	0.28 ***
Number of years using Facebook					0.23	0.16	0.05	0.06	0.16	0.01
Facebook smartphone app use					4.69	1.27	0.11 ***	4.27	1.29	0.10 **
Early maladaptive schemas	Emotional Deprivation							−0.04	0.10	−0.02
	Abandonment							−0.09	0.08	−0.06
	Mistrust/Abuse							0.18	0.10	0.11
	Social Isolation/Alienation							−0.30	0.10	−0.19 **
	Defectiveness/Shame							−0.03	0.11	−0.01
	Failure to Achieve							0.00	0.09	0.01
	Dependence/Incompetence							0.13	0.13	0.07
	Vulnerability to Harm or Illness							0.21	0.11	0.12
	Enmeshment							0.10	0.10	0.05
	Subjugation							0.09	0.13	0.05
	Self-sacrifice							−0.19	0.08	−0.10 *
	Emotional Inhibition							0.03	0.09	0.02
	Unrelenting Standards							−0.08	0.09	−0.04
	Entitlement/Grandiosity							−0.07	0.09	−0.04
	Insufficient Self-Control/Self-Discipline							0.19	0.09	0.11 *
	Approval Seeking							0.41	0.08	0.25 ***
	Pessimism/Negativism							−0.11	0.10	−0.07
	Self-Punitiveness							−0.09	0.09	−0.05
Constant		22.51	3.12		6.86	3.38		4.37	3.39	
R^2^		0.01			0.19			0.30		
F-statistic		2.79			21.26 ***			11.83 ***		
ΔR^2^					0.18 ***			0.11 ***		

*** *p* < 0.001, ** *p* < 0.01, * *p* < 0.05, Note: gender: 0—female, 1—male; Facebook smartphone apps use: 0—No, 1—Yes; smartphone apps use: 0—No, 1—Yes.
